# The use of bisphosphonates does not contraindicate orthodontic and other types of treatment!

**DOI:** 10.1590/2176-9451.19.4.018-026.oin

**Published:** 2014

**Authors:** Alberto Consolaro

**Affiliations:** 1 Full professor, School of Dentistry - University of São Paulo/Bauru and professor at the postgraduate program at the School of Dentistry - University of São Paulo/ Ribeirão Preto.

**Keywords:** Bisphosphonates, Osteomyelitis, Osteonecrosis, Orthodontics, Implants

## Abstract

Bisphosphonates have been increasingly used not only to treat bone diseases as well as conditions such as osteopenia and osteoporosis, but also in oncotherapy. The use of bisphosphonates induces clinicians to fear and care. These reactions are associated with controversy resulting from lack of in-depth knowledge on the mechanisms of action as well as lack of a more accurate assessment of side effects. Scientific and clinical knowledge disclosure greatly contributes to professionals' discernment and inner balance, especially orthodontists. Fear does not lead to awareness. For these reasons, we present an article that focuses on that matter. This article was adapted from different journals of different dental specialties, as mentioned on footnote. There is no scientific evidence demonstrating that bisphosphonates are directly involved with etiopathogenic mechanisms of osteonecrosis and jaw osteomyelitis. Their use is contraindicated and limited in cases of dental treatment involving bone tissue. Nevertheless, such fact is based on professional opinion, case reports, and personal experience or experiment trials with failing methods. Additional studies will always be necessary; however, in-depth knowledge on bone biology is of paramount importance to offer an opinion about the clinical use of bisphosphonates and their further implications. Based on bone biopathology, this article aims at contributing to lay the groundwork for this matter.

## Abstract (pt)

Cada vez mais se usa os bisfosfonatos nos tratamentos de doenças e de estados ósseos, como a osteopenia e osteoporose, assim como nos protocolos oncoterápicos. O uso dos bisfosfonatos induz muitas reações de medo e cuidado, associadas a polêmicas e controvérsias quase sempre resultantes de uma falta de conhecimento mais profundo dos mecanismos de ação e da falta de uma avaliação mais criteriosa de seus efeitos colaterais. A divulgação e o conhecimento dos aspectos científicos e clínicos contribuem, em muito, para o discernimento e tranquilidade dos profissionais, especialmente dos ortodontistas. O medo não resulta em conscientização. Por essas razões, apresentamos alguns artigos sobre o mesmo assunto, semelhantes e adaptados, em revistas voltadas para as diferentes especialidades odontológicas, entre os quais o citado na nota de rodapé. Nos mecanismos etiopatogênicos da osteonecrose e nas osteomielites, nos maxilares, os bisfosfonatos não se encaixam como um dos fatores diretamente envolvidos e com base em evidências científicas. Suas contraindicações e limitações na prática odontológica como fator limitante de alguns tratamentos envolvendo o tecido ósseo estão baseadas, principalmente, em opiniões, casos clínicos e na experiência pessoal ou trabalhos experimentais com algumas falhas nos métodos experimentais. Sempre serão necessários mais estudos, mas um cuidado importante é se aprofundar no conhecimento da biologia óssea para, quando necessário, emitir-se opiniões sobre protocolos de conduta na clínica odontológica quanto ao uso bisfosfonatos e suas implicações. Esse artigo objetiva contribuir na fundamentação de abordagens sobre esse assunto a partir da biopatologia óssea.

Clinical practice must not be limited by fear which usually comes along with ignorance - whether licit, genuine or naive. On the contrary, we have to promote knowledge. Scientific wisdom must be based on scientific evidence rather than opinion, word or faith.^2-5,10^

Personal and clinical experience is valuable when combined with scientific grounds and criteria. Similarly to personal and clinical experience, strictly laboratorial and/or experimental trials should not be considered in isolation either. Combining laboratorial, experimental and clinical outcomes with experience previously described in the literature allows well-grounded procedures to be established, thereby indicating true evolution.

As in any scientific research, results yielded by studies on bisphosphonates must be grounded on well-organized human samples comprising a proper number of subjects, given that there is a wide range of casuistries on human beings subjected to this type of treatment. Should research be conducted with animals, they must be in proper number and subjected to appropriate methods. A researcher, for instance, should not attempt to diagnose osteomyelitis and osteonecrosis in imaging exams of tiny rat mandibles. Diagnosis of bone disease requires a proper number of research animals and microscopic analyses performed by experts. In other words, one cannot reach accurate diagnosis based only on numerical measurements or counts of one or more than one bone component, whether structural or cellular.

Since the first bisphosphonates and bisphosphonate-related treatments (including treatment of osteopenia related to menopause conducted to avoid osteoporosis) arose, we have microscopically investigated its effects on maxillary bone, induced tooth movement, osseointegration and root resorption.^7,8,11,13^

Although they have been widely used for medical purposes, bisphosphonates have caught dentists' attention in the last few years, only.^9,11,13^ Nevertheless, we notice great lack of basic scientific grounds that aid clinicians to understand not only bisphosphonates mechanism of action, but also their clinical applicability, side effects, variation in presentation and dosage. Ignorance leads to myths, controversies and mystification within any knowledge domain. And bisphosphonates are not different.

Some verbalized statements are disturbing, controversial and polemical, yet not well-grounded. For instance:

...patients taking bisphosphonates must not undergo dental treatment involving surgical procedures and bone biology, as it is the case of orthodontic treatment.

...patients taking bisphosphonates do not undergo bone remodeling because bisphosphonates "eliminate" clasts.

...patients taking bisphosphonates on a daily basis run a much higher risk of having jaw osteomyelitis.

Nevertheless, with a view to simply disagreeing with random arguments, we may ask ourselves:

- On which scientific evidence were these clinical decisions based? On which laboratory and clinical investigations published on which journals? Or were they based on case reports and clinical experience assessed by unskilled professionals who are unable to reach accurate, safe and systemic diagnosis of bone disease?
- How many patients take bisphosphonates on a daily basis and undergo dental treatment that includes oral rehabilitation with osseointegrated implants and orthodontic movement, and the clinician did not even hear about? Many patients do not report the use of bisphosphonates during the first interview or are advised not to report it so as to prevent their dentists from feeling scared.
- How many patients who take bisphosphonates actually have problems with rehabilitation and orthodontic treatment? And how many do not?
- What kinds of experience do people who make such statements have from a clinical, laboratory and pharmacological standpoint? On which scientific methods and diagnosis of metabolic, inflammatory bone diseases are they based?

With a view to enhancing understanding about the use of bisphosphonates in human beings as well as their clinical and therapeutic implications, we wrote this article. It addresses the use of bisphosphonates especially with regard to the specificities of Maxillary Orthodontics and Orthopedics.

## Bone remodeling is the target phenomenon of bisphosphonates

Our 206 bones undergo continuous bone remodeling and have from one to three million points of resorption with active BMUs, especially clasts. At these points, moments of bone resorption and neoformation alternate so as to cause skeletal renewal to occur within two to ten years, depending on body site, patient's age and other conditioning factors such as lifestyle and sex.

Should fracture occur in cases of osteopenia and consequent osteoporosis, active BMUs - especially clasts - undergo hyperactivity, acting directly on bone surface and removing mineral ions by releasing acids via active edge. While clasts demineralize, they degrade organic bone matrix by releasing proteolytic enzymes, especially collagenase. The byproduct resulting from bone disassembly (ions, peptides, amino acids) is transposed to peripheral tissue by the clasts and through cytoplasmic vesicles that have their cellular membrane opened towards the opposite side of bone interface. This process of cytoplasmic transport of bone components in the clasts is known as transcytosis.

Bone remodeling does not involve the teeth, even though it occurs at approximately 250 micrometers from the cementum surface. On the root surface, cementoblasts do not have receptors for chemical mediators that promote bone remodeling or turnover such as those of systemic action, parathormone, calcitonin and estrogens; and local action, cytokines, growth factors and arachidonic acid products that act in bone areas associated with cellular stress and inflammation.^2-5^

Once mineral ions and other hard dental tissue components are incorporated, they cannot be naturally removed. Unlike bones, human teeth do not function as or account for a mineral or protein reservoir. Removal of permanent teeth components only occurs in cases of pathological conditions as a result of resorption. Osteopenia and osteoporosis, as well as endocrine system diseases, do not incur in impairment of root surfaces because they are protected by the cementoblast layer.

## Bisphosphonates and their mechanisms of action

Since the 90s, bisphosphonates have been used as medication to control osteopenia and prevent human osteoporosis. They comprise a class of drugs that act on bone metabolism, especially due to its easy and quick combination/bonding with mineral ions, particularly calcium. When ions such as calcium are incorporated into the bone matrix as a result of mineralization, they carry bisphosphonates molecules that become part of the structure naturally reabsorbed during natural skeletal remodeling.

During demineralization, the transport of bisphosphonate-bonded calcium via transcytosis carried out by means of clasts induce biochemical events capable of initiating apoptosis.^2-5^ This process of natural death - in which cells die by structure fragmentation without causing flow of enzymes or molecules that induce inflammation - minimizes bone resorption and slows down the process of remodeling. Thus, bisphosphonates contribute to control bone remodeling or accelerated bone turnover, thereby preventing osteopenia and consequent osteoporosis.

Other mechanisms of action acting simultaneously or parallel to those of bisphosphonates have already been studied and proved.^2-5,7,8,10-14^ The advent of bisphosphonates used to treat osteopenia and osteoporosis promoted an avalanche of publications, including extensive and thorough literature reviews. Every detail of this class of drugs has been properly explored on the book by Bijvoet et al.^1^ In the following paragraphs, we present a synthesis of potential mechanisms of action of bisphosphonates.

The effects of bisphosphonates may occur in three different levels: in the tissue, cell or molecule. Bisphosphonates reduce the extent of absorption regions and the depth of eroded areas as a result of decreasing osteoclastic activity:

- They inhibit recruitment of cells towards bone surface.
- They inhibit cell activity.
- They reduce cell lifetime by inducing apoptosis.
- They affect the process of mineral exchange during bone resorption.

Some of the mechanisms of action that hinder bone resorption are considered controversial, given that they are incompatible with the type of bisphosphonate or bisphosphonate concentration. The effects produced by bisphophonates include: decreased production of lactic acid, inhibition of some lysosomal enzymes, decreased synthesis of prostaglandins as well as decreased multiplication of macrophages.

There is evidence suggesting direct cytotoxic action over clasts. Action exerted over clasts could also result from their inhibition of adhering to bone surfaces. In addition to directly acting over clasts, bisphosphonates also inhibit bone resorption by the indirect action of osteoblasts which interfere in the function of clasts, given that these cells control the active BMUs and recruitment of clasts towards bone surfaces.

Several studies assert that bisphosphonates exert specific action over osteoblasts, particularly clodronate that - in relatively lower doses in comparison to bone resorption reduction or prevention - is able to affect differentiation of osteoblasts, thereby stimulating bone neoformation. Bisphosphonates promote secretion of osteoblasts that inhibit clasts formation and activity.

## Bisphosphonates do not stop bone remodeling, they modulate it!

Bisphosphonates control uncontrolled bone remodeling. They act similarly to some pathological processes such as osteopenia and osteoporosis caused by lack of estrogen (typical of menopause). In these patients, controlled clasts formation and activity reestablishes balance in bone formation and resorption, both of which are essential for the maintenance of bones.

In other words, bisphosphonates aim at restoring bone physiology close to normality. Clinically speaking, bisphosphonates provide patients with comfort and quality of life. They are not anti-remodeling, they modulate and control the process instead.

Researches on induced tooth movement of animals and humans using bisphosphonates were conducted considering the type of bisphosphonates, dosage, administration route, experimental period and model of induced tooth movement. Neither of them assert nor reveal evidence that this type of drug contraindicates orthodontic treatment.^2-8,10-14^ There is no scientific support, methodologies, evidence or outcomes that allow such statement. The same is applied to the process of osseointegration.

## Bisphosphonates and the risk of maxillary osteomyelitis during dental treatment: No biological or scientific evidence

In patients with malignant neoplasm, tumor cells release mediators that simulate the action and effect produced by parathormone on bone tissue. This occurs as a result of molecular similarities among mediators. Thus, patients with malignant neoplasm have extremely accelerated bone resorption and increased serum calcium levels, which is highly life-threatening. For this reason, this condition is known as malignant hypercalcemia.

Bisphosphonates can control uncontrolled bone resorption in oncological patients and, as a result, reduce or remove malignant hypercalcemia. One of the most important effects of bisphosphonates on malignant hypercalcemia is the elimination of intense painful symptoms, typical of this systemic condition.

Patients undergoing treatment of malignant neoplasm make use of several types of medication, including strong antibiotic, analgesic and anti-inflammatory drugs. They also make use of cytostatic and cytotoxic medication that act against malignant cells remaining at the lesion site as well as in other parts of the body, thereby killing them or hindering their proliferation.

Unfortunately, these medications produce antineoplastic side effects that decrease the production of leukocytes, the cells of our immune system. This happens because the bone marrow continuously produces these defense elements at an accelerated pace; however, when in contact with cytostatic and cytotoxic medications, it slows down and strongly impairs patient's immune system. Due to the same reason - low cellular proliferative capacity - regenerative repair processes are compromised.

Many patients undergoing oncological therapy also receive radiotherapy, especially at the primary source of neoplasms; for instance, in case of maxillary neoplasm. During treatment, patients are not able to fully react against minor offending agents, especially microbial ones. Irradiated tissues have even lower capacity, especially maxillary bone tissues which are more susceptible to a particular type of osteomyelitis also known as osteoradionecrosis.

Nevertheless, many non-irradiated patients also often have osteomyelitis resulting from antineoplastic treatment, given that their mouth is more susceptible to receiving a large amount of different species of microorganisms.

Secondary osteomyelitis in patients with malignant neoplasm reflects a condition that has been acknowledged for decades; however, due to well-known frequency and etiology, has been trivialized and under-reported in the literature.

Antineoplastic treatment protocols including the use bisphosphonates caught the attention of some clinicians who began to associate osteomyelitis with bisphosphonates side effects.

Used in isolation, bisphosphonates do not reveal any evidence of susceptibility to osteomyelitis. On the contrary, patient's impaired immune system and tissues with low reactional capacity, as well as the ctytostatic and cytotoxic side effects of the drug, essential for antineoplastic therapy, do reveal evidence of susceptibility to osteomyelitis. In cases of normal patients, bisphosphonates have even lower chances of making individuals susceptible to mandibular or maxillary osteomyelitis. Bisphosphonates do not decrease the efficiency of patient's immune system as inflammation and immunologic response do. Furthermore, not only they do not decrease cell proliferation, but also are not cytotoxic for bone cells.

## When does jaw osteomyelitis occur?

Jaw osteomyelitis occurs almost exclusively under two major clinical conditions:

- In patients with systemic disorders such as anemia, uncontrolled diabetes mellitus, leukemia, alcohol consumption, immunodepression, malignant neoplasm, among others;
- In patients with bone diseases and sclerosing jaw lesions; for instance, cases of Florid Cemento-Osseous Dysplasia and Paget disease.

Innumerous clinicians often seek professional advice on cases of patients using bisphosphonates to modulate bone remodeling of osteopenia and osteoporosis, especially because they "heard rumors" about the fact that these patients would be highly susceptible to osteomyelitis or that their teeth would never move again!

Questions most frequently asked by clinicians about patients using bisphosphonates are as follows:

... *May I move patient's teeth?*

*... May I perform a periodontal surgery?*

*... May I move unerupted teeth by traction?*

*... May I perform implant placement?*

*... May I perform tooth extraction?*

Even though the overall answer is yes, understanding the aforementioned questions is essential for the development of several researches on the influence of bisphosphonates over tooth movement, including clinical and experimental trials as previously suggested.

We have monitored several patients treated at different dental offices where periodontal, surgical, orthodontic and osseointegrated implant rehabilitation treatments are performed without further issues during or after therapy.

In order to reach accurate and complete diagnosis, every case of osteomyelitis must be questioned about the following: Which systemic disorder does the patient carry? The first interview and clinical examination must be supplemented by thorough, accurate complementary exams because systemically healthy patients do not usually have osteomyelitis!

Should healthy patients have osteomyelitis, its causes usually trigger localized bone inflammation restricted to the action site. In these cases, the condition is known as osteitis.

Osteitis may lead to neoformation of reactional bone, in addition to promoting resorption with focal radiolucent areas. We always have to bear in mind the following information.

a) Bisphosphonates are a class of drugs. For this reason, we must choose one and know which one will be used by each patient specifically, since each drug has its own pharmacological specificities. Bisphosphonates used to treat osteopenia and osteoporosis are not usually the same drugs used for oncological patients.

b) Bisphosphonates take longer to react with the organism. First, they need to be incorporated by the bone. Once they are absorbed, their molecules only act over clasts after bone remodeling affects the skeleton.

c) Jaw osteomyelitis requires systemic and local conditions that do not rely on the use of bisphosphonates. Systemically healthy patients scarcely have osteomyelitis. Healthy patients using bisphosphonates to control osteopenia and prevent osteoporosis must be considered healthy and normal from a dental standpoint.

## When does osteonecrosis occur and how is it different from osteomyelitis?

Osteonecrosis conceptually accounts for ***bone tissue and bone marrow death without infection caused by contact with microorganisms, in other words, in the absence of any microbial agent.*** The causes of osteonecrosis of the human skeleton are:

1) Trauma, including bone fracture and surgery.

2) Autograft and allograft areas.

3) Internal and external radiation therapy applied to the affected area.

4) Use of corticosteroids.

5) Focal bone necrosis at different sites; for instance, in the femur head (Legg-Calvé-Perthes disease) and in the navicular bone (Kohler disease).

6) Organ transplantation, especially in patients with persistent hyperparathyroidism even after kidney transplantation.

7) Systemic diseases such as polycythemia, lupus erythematosus, Gaucher disease, sickle-cell disease and gout.

8) Osteochondritis dissecans of unknown etiology with fracture of articular cartilage and the underlying subchondral bone.

9) Emboli capable of leading to bone infarct and thrombosis caused by pressure exerted by local factors such as tumors.

10) Idiopathic factors similarly to what occurs with frequent osteonecrosis in the femur head of alcoholic patients.

Relevant medical literature does not usually comprise articles including bisphosphonates as a cause of osteonecrosis. In many cases, osteonecrosis will be inevitably considered as idiopathic, as it occurs in cases involving the femur head of alcoholic patients.

Osteonecrosis is cured differently in cortex and cancellous bones. Necrosed cancellous bone has its medullary portion gradually replaced by granulation tissue with pluripotent cells necessary for local bone remodeling. Necrotic bone trabeculae may be either reabsorbed by osteoclastic activity or involved by immature or primary bone produced by surrounding granulation tissue. By the end of the process, the trabeculae is remodeled and reshaped by a mechanism of intramembranous ossification.

In the necrotic cortical bone, vascular canals stimulate neovascularization of periosteum and endosteum and, as a result, lead to the formation of cones of bone resorption. Subsequently, the clasts open tunnel-shaped ways in the necrotic compact cortical bone, bringing osteoblasts along and, as a result, leading to bone neoformation. This process is slow and oftentimes deposits lamellar bone.

## When does osteonecrosis change into osteomyelitis?

Pus formation does occur in cases of osteonecrosis in which osteocytes, osteoblasts and clasts of bone surface and medullary space lose vitality. Pus formation occurs as a result of interaction between bacteria and neutrophils. It requires the presence of bacteria, especially *streptococcus* and *staphylococcus*.

Osteonecrosis with pus formation necessarily implies in secondary contamination by pyogenic bacteria, given that osteonecrosis alone is not of microbial origin.

Should there be pus formation, the condition is known as osteomyelitis - even though osteonecrosis may be secondarily infected by bacteria, thereby causing osteomyelitis. Nevertheless, pus is not primarily part of its pathogenesis.

## Alveolar bone density remains stable with the use of bisphosphonates

With a view to assessing alveolar bone density in organisms under action of alendronate bisphosphonates, Santamaria Jr^13^ histomorphometrically assessed 18 male *Wistar *rats equally divided into 1) Control group: without alendronate; 2) Experimental group: receiving 1 mgP/Kg alendronate since intrauterine life and for three months after birth.

Maxillary alveolar bone density was determined in cross section between the roots of murine molars. To this end, a crib with 1200 points was used to establish areas of bone tissue and marrow. Quantification was performed with the aid of Image J 1.34s software. Student's t-test was used to compare control and experimental groups. Significant differences were considered for P ≤ 0.05.

Results revealed no statistically significant differences in alveolar bone density between animals using alendronate and the control group (P = 0.3754). Based on the methods employed, it is reasonable to conclude that bisphosphonate alendronate does not morphologically affect alveolar bone quality, thereby preserving the structural and mechanical tissue characteristics of healthy animals. It is worth noting that the alveolar region assessed herein is the site where teeth are experimentally moved. In other words, it is possible to say that ***orthodontic movement may be naturally planned in organisms under action of bisphosphonates.***

In his PhD dissertation, Santamaria Jr^13^ also reports a case of a 55-year-old patient who had been using bisphosphonate alendronate sodium for 10 years to treat osteopenia and osteoporosis. Later on, the patient was subjected to implant placement and orthodontic treatment with extraction, as well as paraendodontic and periodontal surgery without further complications or clinically observed changes attributed to the use of the medication. By the end of treatment, patient's esthetics and function were restored.

## Requirements to discuss about bisphosphonates

In order to adopt and discuss about bisphosphonates, their indications, contraindications and consequences, a few requirements are necessary:

» In-depth knowledge of bone biology, pathophysiology of clasts, osteoblasts, osteocyte and other structures involved in bone remodeling;
» Extensive and in-depth knowledge of the pharmacological aspects of bisphosphonates so as to avoid the oversimplified belief of "I am against or I am in favor" usually associated with opinion, faith and ideology characteristic of religion and politics.

Both statements are considered necessary not only to establish insights, but also to plan further clinical and research works on this subject that certainly requires additional investigation.

## Final considerations

1) Bisphosphonates do not act to restrain, eliminate or deregulate bone remodeling, but to modulate, control and reestablish balance between bone resorption and neoformation. Should we consider the opposite extreme, cases in which the patient does not use bisphosphonates would be of major concern, given that bone remodeling could be affected.

2) Dentistry - especially Orthodontics and Maxillary Orthopedics - with its surgical treatment plannings, bone graft, dental implants and other bone biology-based procedures, must pursue treatment protocols that take advantage of the pharmacological benefits offered by bisphosphonates to bone remodeling and repair. Some of these advantages could truly benefit patients: Let's research and deepen our knowledge!

3) Bisphosphonates mare pharmacologically different. Their use varies in accordance with indications.

4) The benefits offered by bisphosphonates do not allow generalizations based on specific cases which, most of times, are not deeply studied in clinical, imaginologic, microscopic and etiopathogenic terms.

5) Specific and isolated clinical cases are not capable of determining bisphosphonates contraindication or opinion. Without scientific basis, they may lead to wrong decisions and cause patients undergoing other medical therapies to refuse treatment with bisphosphonates.

Each case must be individually studied and planned without generalizations and with clinical decisions grounded on firm scientific evidence. Nearly all cases presented in the literature as having osteonecrosis and/or osteomyelitis associated with the use of bisphosphonates are not based on severe methodological analysis: There is lack of systemic previous assessment and bone morphology analysis prior to treatment, in addition to the presence of dogmatic assumptions.

## Figures and Tables

**Figure 1:**
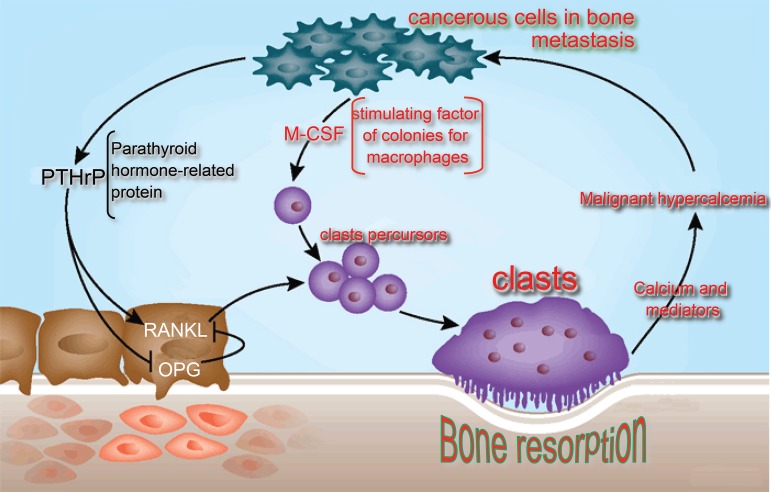
Neoplastic malignant cells release mediators that ease their dissemination in the skeleton. PTHrP is one of these mediators of which function is similar to parathormone, as it induces and stimulates resorption via RANKL so as to accommodate bone metastasis. At the same time, they release M-CSF, a mediator that stimulates the formation of clasts precursors and increases the number of neoplastic malignant cells. This process results in painful malignant hypercalcemia. Bisphosphonates aid to decrease clasts and control painful symptoms. (Source: Consolaro A^3^, 2012, adapted from Roodman GD. Bone-breaking cancer treatment. Nature Med, 13, 25-26, 2007.doi:10.1038/nm0107-25).

**Figure 2:**
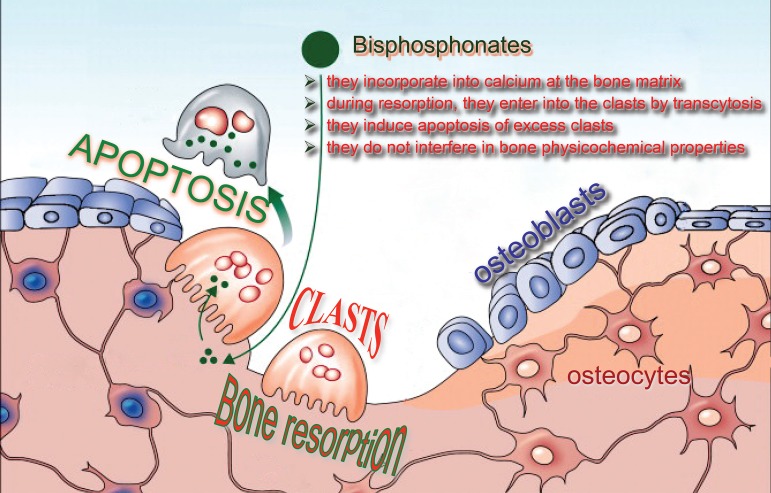
One of the mechanisms of action of bisphosphonates in controlling accelerated bone remodeling consists of controlling the number of clasts, thereby inducing apoptosis when it enters into the medication molecule during bone remodeling caused by transcytosis. Bisphosphonates are easily bonded to calcium and, as a result, incorporated by bone. (Source: Consolaro A^3^, 2012, adapted from Gennari e Bilezikian: Lancet, v.373, Issue 9671, 1225-1226, 2009 doi:10.1016/ S0140-6736(09)60704-22).
